# Incidence of Early Police Contact Among Children With Emerging Mental Health Problems in Australia

**DOI:** 10.1001/jamanetworkopen.2021.12057

**Published:** 2021-06-22

**Authors:** Kimberlie Dean, Tyson Whitten, Stacy Tzoumakis, Kristin R. Laurens, Felicity Harris, Vaughan J. Carr, Melissa J. Green

**Affiliations:** 1 School of Psychiatry, University of New South Wales, Sydney, New South Wales, Australia; 2 Justice Health and Forensic Mental Network, Matraville, New South Wales, Australia; 3 School of Social Sciences, University of Adelaide, Adelaide, South Australia, Australia; 4 School of Criminology and Criminal Justice, Griffith University, Southport, Queensland, Australia; 5 School of Psychology and Counselling, Queensland University of Technology, Brisbane, Queensland, Australia; 6 Institute for Biomedical Innovation, Queensland University of Technology, Brisbane, Queensland, Australia; 7 Neuroscience Research Australia, Sydney, New South Wales, Australia; 8 Department of Psychiatry, Monash University, Melbourne, Victoria, Australia

## Abstract

**Importance:**

In adulthood and adolescence, mental health vulnerability is known to be associated with risk of criminal justice system contact as both a perpetrator and survivor of crime, but whether this association is apparent early in child development is unknown. Prevention of poor outcomes, including repeated contact with the criminal justice system, relies on the identification of vulnerability early in life and at the start of such contact.

**Objective:**

To ascertain whether children with emotional or behavioral problems and general developmental vulnerabilities are at an increased risk of subsequent contact with police as a person of interest, a survivor of crime, or a witness.

**Design, Setting, and Participants:**

This cohort study used routinely collected data from the New South Wales Child Development Study in Australia. The cohort was composed of children who entered full-time schooling in New South Wales in 2009, had complete data for the emotional maturity domain of the Australian Early Development Census, and had no police contact before January 1, 2009. The children in the cohort were followed up until the age of 13 years. Data were analyzed from October 17, 2019, to May 13, 2020.

**Exposures:**

Emotional or behavioral problems and developmental risk profiles derived from the teacher-rated Australian Early Development Census.

**Main Outcomes and Measures:**

Incidence rates of police contact (as a person of interest, survivor of crime, or witness) were derived from the New South Wales Police Force Computerised Operational Policing System.

**Results:**

A total of 79 801 children (40 584 boys [50.9%]; 2009 mean [SD] age, 5.2 [0.37] years) were included. Children with teacher-identified emotional or behavioral problems at school entry had an incidence rate of police contact (for any reason) that was twice that of children without such problems (unadjusted hazard ratio [HR], 2.14; 95% CI, 1.94-2.37). Contact with police as a survivor of crime was most commonly recorded (7350 [9.2%]), but the strength of the association was greatest between emotional or behavioral problems and police contact as a person of interest (unadjusted HR, 4.75; 95% CI, 3.64-6.19). Incidence of police contact as a person of interest was high for children with a pervasive developmental risk profile (unadjusted HR, 13.80; 95% CI, 9.79-19.45).

**Conclusions and Relevance:**

This study found an association of emerging emotional or behavioral problems and developmental vulnerabilities with increased risk of police contact for any reason among young children, suggesting that this well-known association in adults and adolescents can be identified at an earlier developmental stage. These findings support primary and secondary interventions to prevent police contact early in life and to target the earliest contacts with the criminal justice and educational systems.

## Key Points

**Question:**

Are children with mental health problems that were identified at school entry at greater risk of police contact for any reason, ie, as a person of interest, survivor of crime, or witness?

**Findings:**

In this cohort study of 79 801 children followed up from their first year of full-time schooling to 13 years of age, the incidence of early police contact was twice the rate in children with teacher-assessed emotional or behavioral problems at school entry compared with those without such problems. The elevated incidence rates were found for police contact as a person of interest, survivor of crime, or witness.

**Meaning:**

Results of this study suggest that the association between mental illness and increased risk of criminal justice system contact in adulthood and adolescence extends to early childhood.

## Teaser

This cohort study examines the behavioral, developmental, and other factors underlying the first exposure of school-aged children to the criminal justice system in Australia.

## Introduction

Early contact with the criminal justice system is associated with adverse outcomes across the life course in the domains of mental and physical health, educational and occupational attainment, and risk of repeated criminal justice involvement, especially for those who had contact with the justice system after offense perpetration in adolescence and/or adulthood.^1,2^ Those with early contact are also more likely to display a range of antecedent vulnerabilities at the individual, familial, and community levels.^3,4^ The rates of criminal justice system contact as an offender or as a survivor of crime are known to be elevated among adults with mental illness.^5,6,7^ Although this elevated risk is also evident in adolescents with mental disorders,^8^ the extent to which the association between police contact and mental illness exists in childhood is less well understood. The presence of shared risk factors between early mental health problems and early criminal justice system contact (such as trauma,^9,10,11^ socioeconomic disadvantage,^12,13,14^ and parental vulnerabilities^15,16^), at least in the context of offending, supports the likelihood that this association would emerge in childhood.

Individuals with mental illness are known to be at an increased risk of police contact, both as a potential offender and as a survivor of crime, at least during adolescence and adulthood.^7,17,18,19^ The extent to which emerging mental health problems might predispose young children to early police contact has not yet been systematically examined, despite the importance of developing evidence-informed early identification and intervention strategies, including incorporating trauma-informed approaches to system and service responses.^20^

Linking police contact data to a cohort of children who entered school in the Australian state of New South Wales in 2009, a previous study found that almost 1 in 6 children had contact with the police on at least 1 occasion, for any reason, between birth and 13 years of age.^21^ Building on this previous work, we conducted the present study using data from the New South Wales Child Development Study (NSW-CDS). Our aim was to ascertain whether children who were assessed at school entry as having emotional or behavioral problems or general developmental vulnerabilities are at an increased risk of subsequent contact with police as a person of interest, a survivor of crime, or a witness.

## Methods

This cohort study was approved by the New South Wales Population and Health Services Research Ethics Committee, which granted a waiver of the informed consent requirement to enable routinely recorded administrative data to be accessed. We followed the Reporting of Studies Conducted Using Observational Routinely-Collected Health Data (RECORD)^22^ statement.

### Study Population and Exposures

The NSW-CDS^23,24^ cohort includes 87 037 children who entered their first year of full-time schooling in New South Wales in 2009 and had a 2009 Australian Early Development Census (AEDC) record. The AEDC, previously known as the Australian Early Development Index, is a validated and reliable measure of school readiness and childhood development^25,26^ that is completed by teachers for each child shortly after they start school. Record linkage of the NSW-CDS cohort to data from multiple New South Wales government agencies (Department of Education, Department of Communities and Justice, and Ministry of Health) was conducted in 2016,^24^ and police contact data from the New South Wales Police Force were added in 2018 for children born between January 1, 2002, and December 31, 2005. Record linkage was performed by an independent organization (New South Wales Centre for Health Record Linkage) using probabilistic matching of personal identifiers across data sets, such as name, date of birth, address, and sex.^23,24^ The first step in the record linkage involved splitting each data set into 2 to separate the identifiers from the research content variables. The former were used for matching of individuals across data sets, and the latter were used for subsequent data integration to produce a deidentified integrated data set containing all research content variables suitable for analysis. The demographic profile of the NSW-CDS cohort is comparable to that of the New South Wales population.

Childhood development was assessed with the teacher-rated AEDC, which covers 5 broad domains and 16 subdomains of development.^25,26^ The AEDC was adapted from the Early Development Instrument, a tool that has also been extensively validated and tested for reliability, including cross-nationally.^26,27^ The 5 broad domains of the AEDC are social competence, emotional maturity, physical health and well-being, language and cognitive skills (school-based), and communication skills and general knowledge. In this study, we used 3 specific subdomains of the emotional maturity domain (anxious and fearful behavior, aggressive behavior, and hyperactivity and inattention) to index childhood emotional or behavioral problems. Following the standard scoring metric for the AEDC, children with a subdomain score in the bottom 10th percentile of the 2009 national AEDC population distribution were classified as developmentally vulnerable on that subdomain. Although psychometric analyses have demonstrated that the AEDC domains are reliable and valid indicators of child development for Australian children,^26,27^ less is known about the reliability and validity of the subdomain measures when used alone or in other combinations.

Previous work with these NSW-CDS data examined developmental vulnerability across the entire 16 subdomains of the AEDC using latent class analysis and identified 4 specific profiles of overall developmental risk: no risk, mild generalized risk, misconduct risk, and pervasive risk (see Green et al^28^ for the risk profile definitions). These risk profiles have been associated with onset of mental illness in childhood.^29^

### Outcomes and Covariates

Data on children’s contact with police were obtained from the New South Wales Police Force Computerised Operational Policing System, which includes all criminal and noncriminal incidents reported to or detected by New South Wales police since January 1995.^30^ Each incident referred to a unique occurrence that (1) involved the same person; (2) occurred at 1 location; (3) occurred during 1 uninterrupted period; and (4) was categorized as 1 of these police contact types: person of interest, survivor of crime (recorded as “victim” in the police system), or witness. Three covariates were considered to be key potential confounders: (1) child sex; (2) Aboriginal and/or Torres Strait Islander background (both child sex and Aboriginal status were derived from information recorded in several linked data sets); and (3) socioeconomic disadvantage (derived from the Index of Relative Socio-economic Disadvantage of the Socio-Economic Indexes for Areas [SEIFA]), which was based on the child’s residential postcode recorded in the AEDC.^31^

### Statistical Analysis

The NSW-CDS cohort for analysis was limited to the children with complete data for the emotional maturity domain of the AEDC and without police contact before January 1, 2009 (before the year of AEDC administration). Children in the cohort were followed up from January 1, 2009, to the date of their first contact with police for any reason and then separately to the date of their police contact as a person of interest, survivor of crime, or witness, or until the end of the observation period (age 13 years), whichever occurred first. Data were analyzed from October 17, 2019, to May 13, 2020.

The primary analyses involved a series of univariable and multivariable Cox proportional hazards regression models to examine the associations of childhood emotional or behavioral problems overall (ie, vulnerable on at least 1 of the 3 subdomains [anxious and fearful behavior, aggressive behavior, and hyperactivity and inattention]), each of the 3 subdomains, and developmental risk profiles (no risk, mild generalized risk, misconduct risk, and pervasive risk) with incidence of first contact with police for any reason and as a person of interest, survivor of crime, or witness. Secondary subgroup analyses were also conducted to examine boys and girls separately, computing tests of interaction.^32^ For all multivariable analyses, the exposure variables were modeled separately and adjusted for potential confounding factors (SEIFA and Aboriginal and/or Torres Strait Islander background). Variables that violated the assumption of proportionality of hazards were treated as time-varying variables and computed with time-dependent interaction terms.

We reported the hazard ratios (HRs) and 95% CIs as measures of effect and estimate precision. Results were considered statistically significant if the 95% CI did not cross 1.00. Hypotheses were assessed according to a 2-tailed test of significance. The Bonferroni correction was applied to avoid potential type 1 errors associated with multiple comparisons, lowering the significance threshold from *P* < .05 to *P* < .002. Because of the reporting restrictions required to protect privacy, we omitted the results for cells with fewer than 15 children. Analyses were conducted in IBM SPSS Statistics 24 (IBM).

## Results

A total of 79 801 children were included in the sample. Table 1 summarizes the prevalence of emotional or behavioral problems, developmental risk profiles, and covariates for the cohort as a whole (40 584 boys [50.9%] and 39 217 girls [49.1%]) and by police contact type. Between January 1, 2009, (mean [SD] age, 5.2 [0.37] years) and the end of the observation period (13 years of age), 9841 children (12.3%) in the cohort had had at least 1 contact with police after school entry. Contact as a survivor of crime was the most common reason for first police contact (7350 [9.2%]), followed by contact as a person of interest (2731 [3.4%]) and contact as a witness (1833 [2.3%]) (Table 1).

**Table 1.** Prevalence of Emotional or Behavioral Problems, Developmental Risk Profiles, and Covariates by Police Contact Type^a^

Variable	No. (%)				
	Total sample (N = 79 801)	Police contact type			
		Any (n = 9841)	POI (n = 2731)	Survivor of crime (n = 7350)	Witness (n = 1833)
Any emotional or behavioral problem					
No	63 176 (79.2)	6759 (68.7)	1665 (61.0)	5078 (69.1)	1258 (68.6)
Yes	16 625 (20.8)	3082 (31.3)	1066 (39.0)	2272 (30.9)	575 (31.4)
Anxious and fearful behavior					
No	71 313 (89.4)	8486 (86.2)	2334 (85.5)	6316 (85.9)	1566 (85.4)
Yes	8442 (10.6)	1345 (13.7)	392 (14.4)	1028 (14.0)	265 (14.5)
Missing data	46 (<0.1)	<15^b^	<15^b^	<15^b^	<15^b^
Aggressive behavior					
No	73 177 (91.7)	8270 (84.0)	2086 (76.4)	6222 (84.7)	1549 (84.5)
Yes	6603 (8.3)	1565 (15.9)	641 (23.5)	1125 (15.3)	283 (15.4)
Missing data	21 (<0.1)	<15^b^	<15^b^	<15^b^	<15^b^
Hyperactivity and inattention					
No	71 456 (89.5)	8034 (81.6)	2035 (74.5)	6035 (82.1)	1504 (82.1)
Yes	8344 (10.5)	1806 (18.4)	696 (25.5)	1314 (17.9)	329 (17.9)
Missing data	<15^b^	<15^b^	NA	<15^b^	NA
Developmental risk profile					
Pervasive risk	3197 (4.0)	785 (8.0)	330 (12.1)	581 (7.9)	170 (9.3)
Misconduct risk	5368 (6.7)	1166 (11.8)	439 (16.1)	846 (11.5)	197 (10.7)
Mild generalized risk	9015 (11.3)	1466 (14.9)	465 (17.0)	1065 (14.5)	302 (16.5)
No risk	62 206 (78.0)	6422 (65.3)	1497 (54.8)	4856 (66.1)	1164 (63.5)
Missing data	15 (<0.1)	<15^b^	NA	<15^b^	NA
Covariate					
Female child sex	39 217 (49.1)	4599 (46.7)	896 (32.8)	3711 (50.5)	889 (48.5)
Male child sex	40 584 (50.9)	5242 (53.3)	1835 (67.2)	3639 (49.5)	944 (51.5)
Aboriginal and/or Torres Strait Islander background					
No	74 399 (93.2)	8017 (81.5)	1906 (69.8)	6045 (82.2)	1403 (76.5)
Yes	5402 (6.8)	1824 (18.5)	825 (30.2)	1305 (17.8)	430 (23.5)
SEIFA					
Fifth quintile: least disadvantaged	17 896 (22.4)	1149 (11.7)	339 (12.4)	1095 (14.9)	208 (11.3)
Fourth quintile	13 569 (17.0)	1465 (14.9)	347 (12.7)	1096 (14.9)	255 (13.9)
Third quintile	13 705 (17.2)	1673 (17.0)	442 16.2)	1244 (16.9)	310 (16.9)
Second quintile	15 255 (19.1)	2158 (21.9)	624 (22.8)	1609 (21.9)	414 (22.6)
First quintile: most disadvantaged	19 227 (24.1)	3082 (31.3)	976 (35.7)	2297 (31.3)	643 (35.1)
Missing data	149 (0.2)	<15^b^	<15^b^	<15^b^	<15^b^

Abbreviations: NA, not applicable; POI, person of interest; SEIFA, Socio-Economic Indexes for Areas.

^a^ Emotional/behavioral problems were indexed using the anxious and fearful behavior, aggressive behavior, and hyperactivity and inattention subdomains of the emotional maturity domain of the Australian Early Development Census (for all subsequent tables also).

^b^ Because of the reporting restrictions required to protect privacy, we omitted the results for cells with fewer than 15 children.

In unadjusted Cox proportional hazards regression analyses (Table 2), all indices of emotional or behavioral problems vs no problems and all developmental risk profiles vs no risk were associated with a higher rate of police contact for any reason; the latter comparison demonstrated a dose-response pattern of association. Children with teacher-identified emotional or behavioral problems at school entry had an incidence rate of police contact for any reason that was twice that of children without such problems (HR, 2.14; 95% CI, 1.94-2.37).

**Table 2.** Unadjusted Hazard Ratios (HRs) for Associations of Emotional or Behavioral Problems, Developmental Risk Profiles, and Covariates With Police Contact Type

Variable	Police contact type			
	HR (95% CI)			
	Any (n = 9841)	POI (n = 2731)	Survivor of crime (n = 7350)	Witness (n = 1833)
Any emotional or behavioral problem				
No	1 [Reference]	1 [Reference]	1 [Reference]	1 [Reference]
Yes	2.14 (1.94-2.37)^a^	4.75 (3.64-6.19)^a^	2.05 (1.83-2.29)^a^	1.74 (1.58-1.92)
Anxious and fearful behavior				
No	1 [Reference]	1 [Reference]	1 [Reference]	1 [Reference]
Yes	1.85 (1.63-2.11)^a^	1.43 (1.28-1.59)	1.79 (1.55-2.07)^a^	1.44 (1.26-1.63)
Aggressive behavior				
No	1 [Reference]	1 [Reference]	1 [Reference]	1 [Reference]
Yes	2.26 (2.14-2.38)	6.92 (5.19-9.21)^a^	2.11 (1.98-2.24)	2.05 (1.80-2.32)
Hyperactivity and inattention				
No	1 [Reference]	1 [Reference]	1 [Reference]	1 [Reference]
Yes	2.03 (1.93-2.13)	5.63 (4.25-7.47)^a^	1.93 (1.81-2.04)	1.86 (1.65-2.10)
Developmental risk profile				
Pervasive risk	3.47 (3.01-4.01)^a^	13.80 (9.79-19.45)^a^	3.13 (2.66-3.59)^a^	2.81 (2.39-3.30)
Misconduct risk	2.74 (2.47-3.04)^a^	7.61 (5.95-9.74)^a^	2.49 (2.21-2.80)^a^	1.96 (1.69-2.28)
Mild generalized risk	1.78 (1.66-1.91)^a^	3.12 (2.67-3.64)^a^	1.66 (1.53-1.81)^a^	1.76 (1.55-2.00)
No risk	1 [Reference]	1 [Reference]	1 [Reference]	1 [Reference]
Covariate				
Female child sex	1 [Reference]	1 [Reference]	1 [Reference]	1 [Reference]
Male child sex	1.13 (1.09-1.17)^b^	2.09 (1.93-2.26)	0.96 (0.92-1.01)	1.05 (0.96-1.15)
Aboriginal and/or Torres Strait Islander background				
No	1 [Reference]	1 [Reference]	1 [Reference]	1 [Reference]
Yes	4.64 (4.14-5.20)^a^	17.91 (13.67-23.47)^a^	4.16 (3.65-4.74)^a^	4.35 (3.90-4.84)
SEIFA				
Fifth quintile: least disadvantaged	1 [Reference]	1 [Reference]	1 [Reference]	1 [Reference]
Fourth quintile	1.34 (1.26-1.46)	1.35 (1.17-1.57)	1.34 (1.23-1.45)	1.62 (1.25-1.95)
Third quintile	1.54 (1.44-1.65)	1.70 (1.47-1.96)	1.51 (1.39-1.63)	1.95 (1.63-2.32)
Second quintile	1.80 (1.69-1.93)	2.15 (1.89-2.46)	1.76 (1.63-1.90)	2.33 (1.98-2.76)
First quintile: most disadvantaged	2.05 (1.93-2.18)	2.64 (2.34-2.99)	2.00 (1.86-2.15)	2.85 (2.44-3.34)

Abbreviations: POI, person of interest; SEIFA, Socio-Economic Indexes for Areas.

^a^ Time-dependent covariate.

^b^ *P* < .05 but greater than Bonferroni-corrected threshold of *P* < .002.

When first police contact for each of the specific reasons was examined, significant associations were found across the spectrum of emotional or behavioral problems and developmental risk profiles. Particularly large effect sizes were found among children with a pervasive risk profile whose first police contact was as a person of interest (HR, 13.80; 95% CI, 9.79-19.45) (Table 2). Children with any emotional or behavioral problems also had high relative incidence rates for police contact as a person of interest (HR, 4.75; 95% CI, 3.64-6.19).

Relative rates of first police contact for any reason and as a person of interest, survivor of crime, or witness were consistently large for children with Aboriginal and/or Torres Strait Islander background, particularly for first police contact as a person of interest (HR, 17.91; 95% CI, 13.67-23.47) (Table 2). Incidence rates of police contact were higher for boys than girls overall (HR, 1.13; 95% CI, 1.09-1.17) and for contact as a person of interest (HR, 2.09; 95% CI, 1.93-2.26), but no difference by sex was apparent for contact as a survivor of crime (HR, 0.96; 95% CI, 0.92-1.01) or as a witness (HR 1.05; 95% CI, 0.96-1.15). A gradient of association between SEIFA quintile and police contact was apparent, with children born in more disadvantaged areas having higher rates of police contact for any reason (HR, 2.05; 95% CI, 1.93-2.18) compared with those in the least disadvantaged areas (Table 2).

When the analyses were repeated with the cohort stratified by sex (Tables 3 and 4), similar patterns of association were observed for boys. Effect sizes for girls with police contact as a person of interest were lower than for boys (eg, for aggressive behavior: HR, 3.36 [95% CI, 2.77-4.09] vs 5.70 [95% CI, 4.13-7.86]), but the findings were otherwise similar for boys and girls. After adjusting for covariates (Table 5; eTable 1 in the Supplement), all associations were weakened, but most remained significant. Formal tests of interaction by sex in adjusted models indicated significant differences between boys and girls for many of the associations tested (Table 5; eTable 2 in the Supplement).

**Table 3.** Unadjusted Hazard Ratios (HRs) for Associations of Emotional or Behavioral Problems, Developmental Risk Profiles, and Covariates With Police Contact Type for Boys (n = 40 584)

Variable	Police contact type			
	HR (95% CI)			
	Any (n = 5242)	POI (n = 1835)	Survivor of crime (n = 3639)	Witness (n = 944)
Any emotional or behavioral problem				
No	1 [Reference]	1 [Reference]	1 [Reference]	1 [Reference]
Yes	2.21 (1.93-2.52)^a^	4.14 (3.03-5.65)^a^	2.08 (1.78-2.42)^a^	1.67 (1.46-1.90)
Anxious and fearful behavior				
No	1 [Reference]	1 [Reference]	1 [Reference]	1 [Reference]
Yes	1.66 (1.39-1.98)^a^	1.29 (1.14-1.47)	1.66 (1.35-2.03)^a^	1.35 (1.13-1.62)
Aggressive behavior				
No	1 [Reference]	1 [Reference]	1 [Reference]	1 [Reference]
Yes	2.50 (2.14-2.91)^a^	5.70 (4.13-7.86)^a^	2.10 (1.94-2.28)	1.88 (1.61-2.21)
Hyperactivity and inattention				
No	1 [Reference]	1 [Reference]	1 [Reference]	1 [Reference]
Yes	2.30 (1.99-2.66)^a^	4.66 (3.39-6.40)^a^	1.93 (1.79-2.08)	1.90 (1.65-2.20)
Developmental risk profile				
Pervasive risk	3.73 (3.10-4.49)^a^	12.74 (8.57-18.94)^a^	3.14 (2.53-3.90)^a^	3.96 (2.51-6.23)^a^
Misconduct risk	2.78 (2.43-3.18)^a^	6.42 (4.83-8.55)^a^	2.44 (2.09-2.85)^a^	2.13 (1.52-2.98)^a^
Mild generalized risk	1.74 (1.58-1.92)^a^	2.76 (2.29-3.32)^a^	1.57 (1.40-1.76)^a^	1.83 (1.46-2.29)^a^
No risk	1 [Reference]	1 [Reference]	1 [Reference]	1 [Reference]
Aboriginal and/or Torres Strait Islander background				
No	1 [Reference]	1 [Reference]	1 [Reference]	1 [Reference]
Yes	5.61 (4.81-6.54)^a^	27.70 (20.08-38.21)^a^	4.38 (3.64-5.27)^a^	4.29 (3.69-4.99)
SEIFA				
Fifth quintile: least disadvantaged	1 [Reference]	1 [Reference]	1 [Reference]	1 [Reference]
Fourth quintile	1.53 (1.38-1.71)^a^	2.04 (1.63-2.56)^a^	1.46 (1.28-1.66)^a^	1.63 (1.23-2.16)^a^
Third quintile	1.98 (1.74-2.26)^a^	4.00 (2.94-5.45)^a^	1.79 (1.54-2.09)^a^	2.23 (1.59-3.11)^a^
Second quintile	2.57 (2.19-3.02)^a^	7.06 (4.69-10.63)^a^	2.27 (1.89-2.73)^a^	2.67 (1.76-4.04)^a^
First quintile: most disadvantaged	3.39 (2.79-4.11)^a^	12.65 (7.60-21.06)^a^	2.93 (2.35-3.64)^a^	3.91 (2.37-6.46)^a^

Abbreviations: POI, person of interest; SEIFA, Socio-Economic Indexes for Areas.

^a^ Time-dependent covariate.

**Table 4.** Unadjusted Hazard Ratios (HRs) for Associations of Emotional or Behavioral Problems, Developmental Risk Profiles, and Covariates With Police Contact Type for Girls (n = 39 217)

Variable	Police contact type			
	HR (95% CI)			
	Any (n = 4599)	POI (n = 896)	Survivor of crime (n = 3711)	Witness (n = 889)
Any emotional or behavioral problem				
No	1 [Reference]	1 [Reference]	1 [Reference]	1 [Reference]
Yes	2.15 (1.84-2.52)^a^	2.21 (1.91-2.56)	1.81 (1.68-1.95)	1.87 (1.60-2.18)
Anxious and fearful behavior				
No	1 [Reference]	1 [Reference]	1 [Reference]	1 [Reference]
Yes	2.11 (1.75-2.55)^a^	1.55 (1.29-1.88)	1.48 (1.34-1.63)	1.53 (1.27-1.85)
Aggressive behavior				
No	1 [Reference]	1 [Reference]	1 [Reference]	1 [Reference]
Yes	2.42 (2.19-2.68)	3.36 (2.77-4.09)	2.33 (2.09-2.61)	2.50 (2.01-3.12)
Hyperactivity and inattention				
No	1 [Reference]	1 [Reference]	1 [Reference]	1 [Reference]
Yes	2.16 (1.96-2.37)	3.10 (2.58-3.73)	2.17 (1.95-2.41)	1.84 (1.47-2.32)
Developmental risk profile				
Pervasive risk	3.40 (2.67-4.32)^a^	7.06 (3.27-15.21)^a^	2.67 (2.32-3.08)	2.27 (1.67-3.08)
Misconduct risk	2.94 (2.46-3.51)^a^	5.49 (3.21-9.41)^a^	2.49 (2.19-2.82)	2.46 (1.90-3.17)
Mild generalized risk	1.86 (1.66-2.07)^a^	2.84 (2.10-3.85)^a^	1.67 (1.52-1.84)	1.88 (1.56-2.25)
No risk	1 [Reference]	1 [Reference]	1 [Reference]	1 [Reference]
Aboriginal and/or Torres Strait Islander background				
No	1 [Reference]	1 [Reference]	1 [Reference]	1 [Reference]
Yes	3.53 (3.28-3.80)	7.09 (6.17-8.15)	3.22 (2.96-3.50)	4.41 (3.78-5.14)
SEIFA				
Fifth quintile: least disadvantaged	1 [Reference]	1 [Reference]	1 [Reference]	1 [Reference]
Fourth quintile	1.49 (1.33-1.67)^a^	1.35 (1.05-1.75)^b^	1.48 (1.30-1.68)^a^	1.75 (1.35-2.28)
Third quintile	1.84 (1.60-2.11)^a^	1.51 (1.17-1.94)	1.83 (1.57-2.13)^a^	2.01 (1.56-2.60)
Second quintile	2.39 (2.03-2.82)^a^	2.17 (1.73-2.72)	2.35 (1.96-2.82)^a^	2.61 (2.05-3.31)
First quintile: most disadvantaged	2.86 (2.35-3.49)^a^	2.61 (2.11-3.23)	2.80 (2.26-3.48)^a^	2.87 (2.28-3.61)

Abbreviations: POI, person of interest; SEIFA, Socio-Economic Indexes for Areas.

^a^ Time-dependent covariate.

^b^ *P* < .05 but greater than Bonferroni-corrected threshold of *P* < .002.

**Table 5.** Multivariable Hazard Ratios (HRs) for Associations of Emotional or Behavioral Problems and Developmental Risk Profiles With Police Contact Type Stratified by Covariates and SEIFA

Variable	Police contact type, HR (95% CI)			
	Any reason	POI	Survivor of crime	Witness
Total cohort (n = 79 801)				
Any emotional or behavioral problem	1.94 (1.76-2.14)^a^	3.99 (3.06-5.21)^a^	1.87 (1.67-2.09)^a^	1.54 (1.39-1.70)
Anxious and fearful behavior	1.72 (1.51-1.95)^a^	1.28 (1.15-1.42)	1.67 (1.45-1.93)^a^	1.31 (1.15-1.49)
Aggressive behavior	2.07 (1.96-2.19)	5.70 (4.28-7.60)^a^	1.93 (1.81-2.06)	1.79 (1.57-2.03)
Hyperactivity and inattention	1.83 (1.74-1.92)	4.52 (3.40-5.99)^a^	1.74 (1.64-1.85)	1.60 (1.42-1.81)
Developmental risk profile				
Pervasive risk	2.74 (2.37-3.16)^a^	9.47 (6.71-13.38)^a^	2.52 (2.14-2.98)^a^	2.12 (1.78-2.49)
Misconduct risk	2.44 (2.20-2.71)^a^	6.36 (4.97-8.15)^a^	2.25 (1.99-2.53)^a^	1.72 (1.48-2.00)
Mild generalized risk	1.52 (1.42-1.64)^a^	2.45 (2.09-2.86)^a^	1.44 (1.33-1.57)^a^	1.44 (1.27-1.64)
No risk	1 [Reference]	1 [Reference]	1 [Reference]	1 [Reference]
Boys only (n = 40 584)				
Any emotional or behavioral problem	1.99 (1.74-2.27)^a^	3.46 (2.53-4.72)^a^	1.64 (1.53-1.75)	1.47 (1.29-1.68)
Anxious and fearful behavior	1.53 (1.28-1.83)^a^	1.17 (1.02-1.33)^b^	1.55 (1.26-1.90)^a^	1.23 (1.03-1.48)
Aggressive behavior	1.99 (1.86-2.13)	4.72 (3.41-6.52)^a^	1.93 (1.78-2.09)	1.64 (1.40-1.93)
Hyperactivity and inattention	2.04 (1.76-2.36)	3.79 (2.76-5.22)^a^	1.76 (1.64-1.90)	1.66 (1.44-1.93)
Developmental risk profile				
Pervasive risk	3.00 (2.49-3.61)^a^	9.01 (6.05-13.41)^a^	2.58 (2.08-3.21)^a^	2.42 (1.99-2.95)
Misconduct risk	2.47 (2.16-2.82)^a^	5.37 (4.03-7.15)^a^	2.20 (1.89-2.58)^a^	1.60 (1.32-1.94)
Mild generalized risk	1.49 (1.36-1.64)^a^	2.18 (1.80-2.62)^a^	1.37 (1.22-1.54)^a^	1.39 (1.16-1.67)
No risk	1 [Reference]	1 [Reference]	1 [Reference]	1 [Reference]
Girls only (n = 39 217)				
Any emotional or behavioral problem	1.95 (1.67-2.27)^a^	1.88 (1.63-2.18)	1.66 (1.54-1.79)	1.66 (1.40-1.96)
Anxious and fearful behavior	1.96 (1.63-2.37)^a^	1.38 (1.14-1.66)	1.38 (1.26-1.52)	1.51 (1.23-1.85)
Aggressive behavior	2.20 (1.99-2.43)	2.80 (2.30-3.41)	2.13 (1.90-2.38)	2.05 (1.61-2.61)
Hyperactivity and inattention	1.87 (1.70-2.06)	2.39 (1.99-2.89)	1.90 (1.71-2.11)	1.42 (1.10-1.83)
Developmental risk profile				
Pervasive risk	2.55 (2.00-3.24)^a^	2.45 (1.90-3.17)	2.10 (1.82-2.43)	1.60 (1.18-2.19)
Misconduct risk	2.59 (2.16-3.10)^a^	2.99 (2.38-3.74)	2.26 (1.99-2.57)	2.10 (1.63-2.72)
Mild generalized risk	1.58 (1.41-1.76)^a^	1.79 (1.50-2.14)	1.45 (1.32-1.59)	1.52 (1.26-1.82)
No risk	1 [Reference]	1 [Reference]	1 [Reference]	1 [Reference]

Abbreviation: POI, person of interest.

^a^ Time-dependent covariate.

^b^ *P* < .05 but greater than Bonferroni-corrected threshold of *P* < .002.

## Discussion

In this study of children commencing their first year of full-time education in New South Wales in 2009, emerging emotional or behavioral problems and general developmental vulnerabilities identified by teachers at school entry were found to be associated with an increased incidence of early police contact for any reason. Among children who had at least 1 contact with police by 13 years of age, almost one-third were found to have at least 1 emotional or behavioral problem at school entry, with the incidence rate being twice that of children without such problems. Incidence rates of police contact over the follow-up period were also higher for those with specific developmental risk profiles, particularly children with a pervasive risk profile. The elevated incidence rates of police contact were confirmed in analyses that focused on specific types of contact, including contact as a person of interest, survivor of crime, or witness, and in analyses that adjusted for key sociodemographic factors. Significant differences in the associations of emotional or behavioral problems and developmental risk profiles with incidence of police contact were found when boys and girls were compared.

Overall, the findings from this study confirmed that the association between mental health problems and risk of contact with the criminal justice system, in the context of both being the offender and experiencing a crime, can be ascertained at an early developmental stage. The findings extended the existing knowledge of this association, which is well established later in life,^7,8,33^ to both an earlier developmental period and to police contact for a range of reasons. These findings suggest the need to direct preventive interventions much earlier in life and to consider early service or system contact (ie, first police contact and first school contact) as a specific target for such interventions. Opportunities exist for systematically identifying at-risk children and families and for implementing targeted interventions, including a trauma-informed response to vulnerabilities that were detected.^20^ A recent systematic review of trauma-informed interventions for young people who were involved in the criminal justice system highlighted a number of promising programs as well as a gap in the evidence of the implications of these treatments, such as for subsequent criminal justice system contact.^34^ The extent to which these programs might be appropriate and beneficial for young children who are making their first contact with the justice and education systems is also untested.

Although an increased incidence of police contact was seen across all indices of emotional or behavioral problems and developmental risk profiles that were evident at school entry, as well as for all types of police contact, we found strong associations for a number of specific combinations. Being a survivor of crime was the most common reason that children had contact with the police, but the strongest associations were found for police contact as a person of interest; for example, children with vulnerability on the aggressive behavior subdomain of the AEDC had an incident rate of person-of-interest police contact that was almost 7 times higher than the rate for children without such problems. These findings are consistent with the results of studies into life-course risk factors for criminal justice system contact, particularly the well-established finding that the risk of perpetuating a crime later in life is higher among those with identified behavioral problems in childhood.^35^

In the current study, the consideration of emotional or behavioral problems alongside the broader spectrum of developmental risk profiles (these problems were derived from subdomains that also contributed to risk profiles) also highlighted the nonspecificity of associations between vulnerability types and risk of criminal justice system contact, at least in early life. Although the pervasive nature of the developmental vulnerabilities identified may reflect some underlying social and other disadvantages or adversity, the increased risk of police contact for those in the pervasive risk group was not fully accounted for by confounding from the sociodemographic indicators of disadvantage that we examined. Approaches for identifying children who are at risk of early police contact may need to consider the patterns of developmental vulnerability across multiple domains rather than narrowing down to only those children with emotional or behavioral problems.

### Sex and Aboriginal and/or Torres Strait Islander Status

In this cohort study, boys were only slightly more likely to have any police contact after school entry and until 13 years of age, although they were twice as likely to have contact as a person of interest. When stratified by sex, the associations followed a pattern similar to that found for the overall cohort, but some significant differences were observed in the strength of the associations for boys vs for girls. Thus, as a number of previous studies have reported,^36,37,38^ we observed key differences in the association of specific factors with risk of police contact between boys and girls. Further research is needed to elucidate the differences in longitudinal patterns and factors associated with criminal justice system contact; however, evidence is already emerging to support the benefits of a gender-specific approach to primary and secondary prevention of criminal justice system contact.^39,40^

Individuals with Aboriginal and/or Torres Strait Islander background in Australia experience high relative rates of criminal justice system contact, including incarceration.^41^ Thus, it appears that the high relative rates of police contact experienced by the adult and adolescent Aboriginal and/or Torres Strait Islander population are also seen in childhood when first contacts with the police occur. First contact with police may present an opportunity to identify and divert vulnerable, young indigenous people, particularly those with emerging emotional or behavioral problems and developmental vulnerabilities, but these diversion approaches need to be culturally informed and appropriate. A recent review of available research and diversionary programs proposed 9 principles of good practice in the diversion of young Aboriginal and/or Torres Strait Islanders away from the criminal justice system.^42^ These principles included self-determination, cultural safety or security, and family-centered holistic support.^42^

### Strengths and Limitations

This study has several strengths. It used a large and representative sample, avoided sampling and information biases that are present in many longitudinal studies, and included a wide range of available information in the analyses. Using police data, rather than other sources of criminal justice system contact information, enabled us to examine the earliest criminal justice system contacts that might occur in childhood as well as contacts for a range of reasons and not only potential offending behaviors.

This study has a number of limitations. First, the limitations inherent in record-linkage studies using administrative data are important to consider, including the absence of variables of potential interest and the reliance on outcome measures to reach a threshold for service contact. In addition, the measures of emotional or behavioral problems and the developmental risk profiles were derived from a single assessment completed by teachers shortly after school entry. Second, although the original cohort has been shown to be representative of the source population in the Australian state of New South Wales,^24^ the extent to which the findings are generalizable to other jurisdictions, within Australia and beyond, is uncertain. In particular, criminal justice policies and practices that affect patterns of police contact may differ even in otherwise comparable populations. Furthermore, the nature and extent of ethnic diversity in the current sample may differ from those in other populations, and both teacher assessment of child development and policing practices may be affected by the ethnic background of children, including Aboriginal and/or Torres Strait Islander children.

Third, we chose to examine the incidence of all types of police contact rather than focus on either potential to offend or to experience a crime, which may present challenges for interpretation given that most relevant research does not take this approach. Particularly in early childhood, the risk of contact with the police is likely to reflect underlying developmental vulnerabilities in both groups, and it is the nature of this vulnerability that is the focus of the current study. In addition, there is considerable overlap between individuals with criminal justice system contact because they committed an offense and those whose contact was related to experiencing a crime, and this overlap may be particularly true early in life. A previous analysis of the NSW-CDS sample found that one-fifth of those with any police contact in childhood had contact for more than 1 reason.^21^

## Conclusions

This large longitudinal cohort study demonstrated that the well-known association between mental health problems and contact with the criminal justice system in adults and adolescents can be identified at an earlier developmental stage and extends to police contact for any reason. These findings provide support for primary and secondary interventions to prevent police contact early in life and to target the earliest contacts with the criminal justice and educational systems.

## Notes

Supplement. **eTable 1.** Multivariable Hazard Ratios (HR; with 95% Confidence Intervals [CI]) for Associations Between Emotional/Behavioural Problems, Developmental Risk Profiles and Children’s Police Contact Type, Stratified by Child Sex, and Adjusted for Aboriginal/Torres Strait Islander Background, and SEIFA (With Estimates for All Variables Included in Models Provided) **eTable 2.** Formal Testing of Potential Interaction by Child Sex in Multivariable Models
