# Supplementary material for: Incidence of Early Police Contact Among Children With Emerging Mental Health Problems in Australia
Source: JAMA Netw Open. 2021 Jun 22;4(6):e2112057. doi: 10.1001/jamanetworkopen.2021.12057 (PMC8220465; doi:10.1001/jamanetworkopen.2021.12057)
Supplement: Supplement. — eTable 1. Multivariable Hazard Ratios (HR; with 95% Confidence Intervals [CI]) for Associations Between Emotional/Behavioural Problems, Developmental Risk Profiles and Children’s Police Contact Type, Stratified by Child Sex, and Adjusted for Aboriginal/Torres Strait Islander Background, and SEIFA (With Estimates for All Variables Included in Models Provided) eTable 2. Formal Testing of Potential Interaction by Child Sex in Multivariable Models [file jamanetwopen-e2112057-s001.pdf]

Supplementary Online Content

Dean K, Whitten T, Tzoumakis S, et al. Incidence of early police contact among children with emerging mental health problems in Australia. *JAMA Netw Open*. 2021;4(6):e2112057. doi:10.1001/jamanetworkopen.2021.12057

eTable 1. Multivariable Hazard Ratios (HR; with 95% Confidence Intervals [CI]) for Associations Between Emotional/Behavioural Problems, Developmental Risk Profiles and Children's Police Contact Type, Stratified by Child Sex, and Adjusted for Aboriginal/Torres Strait Islander Background, and SEIFA (With Estimates for All Variables Included in Models Provided)

eTable 2. Formal Testing of Potential Interaction by Child Sex in Multivariable Models

This supplementary material has been provided by the authors to give readers additional information about their work.

© 2021 Dean K et al. *JAMA Network Open.*

eTable 1. Multivariable Hazard Ratios (HR; with 95% Confidence Intervals [CI]) for Associations Between Emotional/Behavioural Problems, Developmental Risk Profiles and Children's Police Contact Type, Stratified by Child Sex, and Adjusted for Aboriginal/Torres Strait Islander Background, and SEIFA (With Estimates for All Variables Included in Models Provided)

SAMPLE			ANY CONTACT	POI CONTACT	VICTIM CONTACT	WITNESS CONTACT
			HR (95% CI)	HR (95% CI)	HR (95% CI)	HR (95% CI)
TOTAL COHORT (n=79,801)	Any Emotional/Behavioural Problem		1.94 (1.76-2.14)[t]	3.99 (3.06-5.21)[t]	1.87 (1.67-2.09)[t]	1.54 (1.39-1.70)
	Aboriginal/Torres Strait Islander		3.19 (3.03-3.36)	5.37 (4.94-5.84)	2.87 (2.70-3.05)	3.65 (3.26-4.07)
	SEFIA quintile	1	1.69 (1.59-1.80)	1.86 (1.64-2.11)	1.67 (1.56-1.80)	2.25 (1.92-2.64)
		2	1.57 (1.47-1.68)	1.66 (1.45-1.90)	1.55 (1.44-1.68)	1.98 (1.67-2.34)
		3	1.40 (1.31-1.51)	1.42 (1.23-1.64)	1.38 (1.27-1.50)	1.74 (1.46-2.08)
		4	1.27 (1.18-1.36)	1.18 (1.02-1.38)*	1.26 (1.16-1.37)	1.49 (1.24-1.79)
		5	1.00 reference	1.00 reference	1.00 reference	1.00 reference
	Anxious and fearful behaviour		1.72 (1.51-1.95)[t]	1.28 (1.15-1.42)	1.67 (1.45-1.93)[t]	1.31 (1.15-1.49)
	Aboriginal/Torres Strait Islander background		3.30 (3.13-3.48)	5.72 (5.26-6.22)	2.97 (2.79-3.16)	3.76 (3.36-4.19)
	SEFIA quintile	1	1.73 (1.62-1.84)	1.93 (1.70-2.19)	1.71 (1.59-1.83)	2.29 (1.96-2.69)
		2	1.60 (1.50-1.71)	1.72 (1.51-1.97)	1.58 (1.46-1.70)	2.00 (1.70-2.37)
		3	1.42 (1.33-1.53)	1.46 (1.27-1.68)	1.40 (1.29-1.52)	1.76 (1.48-2.10)
		4	1.27 (1.18-1.37)	1.20 (1.03-1.39)*	1.26 (1.16-1.37)	1.49 (1.24-1.80)
		5	1.00 reference	1.00 reference	1.00 reference	1.00 reference
	Aggressive behaviour		2.07 (1.96-2.19)	5.70 (4.28-7.60)[t]	1.93 (1.81-2.06)	1.79 (1.57-2.03)
	Aboriginal/Torres Strait Islander background		3.22 (3.05-3.39)	5.39 (4.96-5.86)	2.89 (2.72-3.08)	3.66 (3.28-4.09)
	SEFIA quintile	1	1.71 (1.60-1.82)	1.88 (1.65-2.13)	1.69 (1.57-1.82)	2.28 (1.94-2.67)
		2	1.59 (1.49-1.71)	1.70 (1.49-1.94)	1.58 (1.46-1.70)	2.00 (1.69-2.37)
		3	1.42 (1.32-1.52)	1.43 (1.24-1.65)	1.39 (1.29-1.51)	1.76 (1.47-2.10)

© 2021 Dean K et al. *JAMA Network Open.*

		4	1.28 (1.19-1.37)	1.19 (1.03-1.39)[*]	1.27 (1.16-1.38)	1.50 (1.25-1.80)
		5	1.00 reference	1.00 reference	1.00 reference	1.00 reference
	Hyperactivity and inattention		1.83 (1.74-1.92)	4.52 (3.40-5.99)[t]	1.74 (1.64-1.85)	1.60 (1.42-1.81)
	Aboriginal/Torres Strait Islander background		3.19 (3.03-3.36)	5.35 (4.92-5.82)	2.88 (2.71-3.06)	3.66 (3.28-4.09)
	SEFIA quintile	1	1.70 (1.60-1.82)	1.87 (1.65-2.12)	1.68 (1.57-1.81)	2.27 (1.94-2.66)
		2	1.58 (1.48-1.69)	1.68 (1.47-1.92)	1.57 (1.45-1.69)	1.99 (1.69-2.36)
		3	1.41 (1.32-1.52)	1.44 (1.25-1.65)	1.39 (1.28-1.51)	1.76 (1.47-2.09)
		4	1.27 (1.18-1.37)	1.19 (1.02-1.38)[*]	1.26 (1.16-1.37)	1.50 (1.25-1.80)
		5	1.00 reference	1.00 reference	1.00 reference	1.00 reference
	Developmental Risk Profiles	Pervasive risk	2.74 (2.37-3.16)[t]	9.47 (6.71-13.38)[t]	2.52 (2.14-2.98)[t]	2.12 (1.78-2.49)
		Misconduct risk	2.44 (2.20-2.71)[t]	6.36 (4.97-8.15)[t]	2.25 (1.99-2.53)[t]	1.72 (1.48-2.00)
		Mild risk	1.52 (1.42-1.64)[t]	2.45 (2.09-2.86)[t]	1.44 (1.33-1.57)[t]	1.44 (1.27-1.64)
		No risk	1.00 reference	1.00 reference	1.00 reference	1.00 reference
	Aboriginal/Torres Strait Islander background		3.08 (2.92-3.24)	5.04 (4.63-5.49)	2.78 (2.61-2.96)	3.48 (3.11-3.89)
	SEFIA quintile	1	1.64 (1.54-1.75)	1.75 (1.55-1.99)	1.63 (1.51-1.75)	2.16 (1.85-2.54)
		2	1.55 (1.45-1.65)	1.61 (1.41-1.85)	1.53 (1.42-1.66)	1.93 (1.64-2.29)
		3	1.38 (1.29-1.49)	1.39 (1.20-1.60)	1.36 (1.26-1.48)	1.71 (1.44-2.04)
		4	1.26 (1.29-1.49)	1.17 (1.01-1.36)[*]	1.25 (1.15-1.36)	1.48 (1.23-1.78)
		5	1.00 reference	1.00 reference	1.00 reference	1.00 reference
BOYS ONLY (n=40,584)	Any emotional/Behavioural Problem		1.99 (1.74-2.27)[t]	3.46 (2.53-4.72)[t]	1.64 (1.53-1.75)	1.47 (1.29-1.68)
	Aboriginal/Torres Strait Islander background		3.30 (3.07-3.54)	5.22 (4.70-5.79)	2.95 (2.71-3.21)	3.57 (3.06-4.17)
	SEFIA quintile	1	1.64 (1.50-1.79)	1.91 (1.64-2.23)	1.56 (1.41-1.73)	2.26 (1.82-2.81)
		2	1.49 (1.46-1.63)	1.68 (1.43-1.98)	1.42 (1.27-1.58)	1.78 (1.41-2.25)
		3	1.36 (1.24-1.50)	1.50 (1.26-1.78)	1.30 (1.16-1.45)	1.69 (1.33-2.15)
		4	1.25 (1.13-1.38)	1.20 (0.99-1.45)	1.22 (1.09-1.37)	1.39 (1.08-1.80)[*]
		5	1.00 reference	1.00 reference	1.00 reference	1.00 reference
	Anxious and fearful behaviour		1.53 (1.28-1.83)[t]	1.17 (1.02-1.33)[*]	1.55 (1.26-1.90)[t]	1.23 (1.03-1.48)

© 2021 Dean K et al. *JAMA Network Open.*

Aboriginal/Torres Strait Islander background		3.44 (3.20-3.69)	5.58 (5.03-6.19)	3.07 (2.82-3.35)	3.68 (3.15-4.30)
SEFIA quintile	1	1.68 (1.54-1.83)	1.99 (1.71-2.32)	1.60 (1.44-1.77)	2.31 (1.86-2.87)
	2	1.52 (1.39-1.67)	1.75 (1.49-2.06)	1.45 (1.30-1.62)	1.82 (1.44-2.30)
	3	1.38 (1.26-1.52)	1.54 (1.30-1.83)	1.32 (1.17-1.48)	1.71 (1.34-2.18)
	4	1.26 (1.14-1.39)	1.22 (1.01-1.46)*	1.23 (1.09-1.38)	1.39 (1.08-1.80)*
	5	1.00 reference	1.00 reference	1.00 reference	1.00 reference
Aggressive behaviour		1.99 (1.86-2.13)	4.72 (3.41-6.52)[t]	1.93 (1.78-2.09)	1.64 (1.40-1.93)
Aboriginal/Torres Strait Islander background		3.32 (3.09-3.56)	5.20 (4.69-5.77)	2.96 (2.71-3.22)	3.57 (3.06-4.17)
SEFIA quintile	1	1.64 (1.51-1.79)	1.91 (1.64-2.23)	1.57 (1.41-1.73)	2.27 (1.83-2.83)
	2	1.51 (1.37-1.65)	1.71 (1.45-2.01)	1.44 (1.29-1.60)	1.81 (1.43-2.28)
	3	1.37 (1.24-1.50)	1.50 (1.26-1.79)	1.30 (1.16-1.46)	1.70 (1.33-2.16)
	4	1.25 (1.13-1.38)	1.20 (0.99-1.44)	1.22 (1.09-1.37)	1.39 (1.07-1.79)*
	5	1.00 reference	1.00 reference	1.00 reference	1.00 reference
Hyperactivity and inattention		2.04 (1.76-2.36)[t]	3.79 (2.76-5.22)[t]	1.76 (1.64-1.90)	1.66 (1.44-1.93)
Aboriginal/Torres Strait Islander background		3.31 (3.08-3.55)	5.23 (4.72-5.80)	2.96 (2.71-3.22)	3.56 (3.05-4.15)
SEFIA quintile	1	1.65 (1.51-1.79)	1.93 (1.66-2.25)	1.57 (1.41-1.73)	2.26 (1.82-2.81)
	2	1.49 (1.36-1.63)	1.68 (1.43-1.98)	1.42 (1.27-1.59)	1.78 (1.41-2.25)
	3	1.37 (1.24-1.51)	1.51 (1.27-1.80)	1.30 (1.16-1.46)	1.69 (1.33-2.16)
	4	1.25 (1.13-1.38)	1.21 (1.00-1.45)*	1.22 (1.09-1.38)	1.39 (1.08-1.80)*
	5	1.00 reference	1.00 reference	1.00 reference	1.00 reference
Developmental Risk Profiles	Pervasive risk	3.00 (2.49-3.61)[t]	9.01 (6.05-13.41)[t]	2.58 (2.08-3.21)[t]	2.42 (1.99-2.95)
	Misconduct risk	2.47 (2.16-2.82)[t]	5.37 (4.03-7.15)[t]	2.20 (1.89-2.58)[t]	1.60 (1.32-1.94)
	Mild risk	1.49 (1.36-1.64)[t]	2.18 (1.80-2.62)[t]	1.37 (1.22-1.54)[t]	1.39 (1.16-1.67)
	No risk	1.00 reference	1.00 reference	1.00 reference	1.00 reference
Aboriginal/Torres Strait Islander background		3.21 (2.99-3.44)	4.98 (4.48-5.52)	2.87 (2.63-3.13)	3.38 (2.89-3.95)
SEFIA quintile	1	1.59 (1.46-1.73)	1.82 (1.56-2.12)	1.51 (1.37-1.68)	2.14 (1.72-2.67)

© 2021 Dean K et al. *JAMA Network Open.*

		2	1.46 (1.33-1.60)	1.63 (1.38-1.92)	1.40 (1.25-1.56)	1.72 (1.36-2.18)
		3	1.34 (1.22-1.47)	1.46 (1.23-1.73)	1.28 (1.14-1.43)	1.64 (1.29-2.09)
		4	1.24 (1.12-1.37)	1.18 (0.98-1.42)[*]	1.21 (1.08-1.36)	1.37 (1.06-1.76)[*]
		5	1.00 reference	1.00 reference	1.00 reference	1.00 reference
GIRLS ONLY (n=39,217)	Any emotional/Behavioural Problem		1.95 (1.67-2.27)[t]	1.88 (1.63-2.18)	1.66 (1.54-1.79)	1.66 (1.40-1.96)
	Aboriginal/Torres Strait Islander background		3.08 (2.85-3.32)	6.04 (5.24-6.98)	2.79 (2.56-3.04)	3.29 (2.70-3.88)
		1	1.76 (1.60-1.93)	1.77 (1.42-2.20)	1.80 (1.62-2.00)	1.76 (1.56–1.95)
		2	1.67 (1.51-1.84)	1.65 (1.31-2.08)	1.70 (1.52-1.89)	1.62 (1.44-1.79)
	SEFIA quintile	3	1.45 (1.31-1.61)	1.26 (0.98-1.62)	1.47 (1.31-1.66)	1.42 (1.28-1.65)
		4	1.29 (1.16-1.44)	1.17 (0.90-1.51)	1.30 (1.15-1.46)	1.18 (1.08-1.35)[*]
		5	1.00 reference	1.00 reference	1.00 reference	1.00 reference
	Anxious and fearful behaviour		1.96 (1.63-2.37)[t]	1.38 (1.14-1.66)	1.38 (1.26-1.52)	1.51 (1.23-1.85)
	Aboriginal/Torres Strait Islander background		3.17 (2.93-3.42)	6.28 (5.44-7.24)	2.87 (2.63-3.13)	2.79 (2.57-3.07)
		1	1.79 (1.63-1.96)	1.81 (1.46-2.26)	1.83 (1.64-2.03)	1.80 (1.60-2.06
		2	1.69 (1.53-1.86)	1.68 (1.33-2.11)	1.71 (1.54-1.91)	1.69 (1.45-1.98)
	SEFIA quintile	3	1.46 (1.32-1.62)	1.27 (0.99-1.64)	1.48 (1.32-1.67)	1.47 (1.29-1.70)
		4	1.30 (1.17-1.44)	1.18 (0.91-1.53)	1.30 (1.16-1.57)	1.22 (1.02-1.52)[*]
		5	1.00 reference	1.00 reference	1.00 reference	1.00 reference
	Aggressive behaviour		2.20 (1.99-2.43)	2.80 (2.30-3.41)	2.13 (1.90-2.38)	2.05 (1.61-2.61)
	Aboriginal/Torres Strait Islander background		3.11 (2.88-3.35)	6.09 (5.27-7.03)	2.82 (2.59-3.08)	2.80 (2.55-3.12)
		1	1.78 (1.62-1.96)	1.80 (1.45-2.24)	1.83 (1.65-2.03)	1.80 (1.42-2.27)
		2	1.70 (1.54-1.88)	1.69 (1.35-2.13)	1.73 (1.55-1.93)	1.68 (1.30-2.17)
	SEFIA quintile	3	1.48 (1.33-1.64)	1.28 (0.99-1.65)	1.50 (1.33-1.68)	1.40 (1.24-1.58)
		4	1.31 (1.18-1.46)	1.18 (0.92-1.53)	1.31 (1.17-1.48)	1.23 (1.08-1.45)[*]
		5	1.00 reference	1.00 reference	1.00 reference	1.00 reference
	Hyperactivity and inattention		1.87 (1.70-2.06)	2.39 (1.99-2.89)	1.90 (1.71-2.11)	1.42 (1.10-1.83)

© 2021 Dean K et al. *JAMA Network Open.*

Aboriginal/Torres Strait Islander background		3.08 (2.85-3.32)	5.97 (5.17-6.90)	2.79 (2.56-3.04)	2.99 (2.41-3.58)
SEFIA quintile	1	1.77 (1.62-1.95)	1.78 (1.43-2.21)	1.81 (1.63-2.01)	2.18 (1.70-2.73)
	2	1.70 (1.54-1.87)	1.69 (1.34-2.12)	1.72 (1.54-1.92)	2.10 (1.62-2.69)
	3	1.46 (1.32-1.62)	1.27 (0.99-1.64)	1.48 (1.32-1.67)	1.77 (1.36-2.29)
	4	1.29 (1.16-1.44)	1.17 (0.90-1.51)	1.30 (1.15-1.46)	1.58 (1.22-2.06)
	5	1.00 reference	1.00 reference	1.00 reference	1.00 reference
Developmental Risk Profiles	Pervasive risk	2.55 (2.00-3.24)[t]	2.45 (1.90-3.17)	2.10 (1.82-2.43)	1.60 (1.18-2.19)
	Misconduct risk	2.59 (2.16-3.10)[t]	2.99 (2.38-3.74)	2.26 (1.99-2.57)	2.10 (1.63-2.72)
	Mild risk	1.58 (1.41-1.76)[t]	1.79 (1.50-2.14)	1.45 (1.32-1.59)	1.52 (1.26-1.82)
	No risk	1.00 reference	1.00 reference	1.00 reference	1.00 reference
Aboriginal/Torres Strait Islander background		2.95 (2.73-3.18)	5.63 (4.87-6.51)	2.87 (2.63-3.13)	3.59 (3.06-4.21)
SEFIA quintile	1	1.70 (1.55-1.87)	1.68 (1.35-2.09)	1.51 (1.37-1.68)	2.18 (1.73-2.76)
	2	1.65 (1.50-1.82)	1.61 (1.28-2.03)	1.40 (1.25-1.56)	2.17 (1.71-2.77)
	3	1.44 (1.29-1.59)	1.24 (0.96-1.59)	1.28 (1.14-1.43)	1.79 (1.39-2.32)
	4	1.29 (1.16-1.43)	1.16 (0.90-1.51)	1.21 (1.08-1.36)	1.60 (1.23-2.09)
	5	1.00 reference	1.00 reference	1.00 reference	1.00 reference

[t]time-dependent covariate; POI = person-of-interest; SEIFA = Socio-Economic Index for Areas
[*]*p*<.05 but greater than Bonferroni corrected threshold of *p*<.002.
Emotional/behavioural problems were indexed using the anxious and fearful behaviour, aggressive behaviour, and hyperactivity and inattention subdomains of the emotional maturity domain of the Australian Early Development Census

© 2021 Dean K et al. *JAMA Network Open.*

eTable2. Formal Testing of Potential Interaction by Child Sex in Multivariable Models

VARIABLE		ANY CONTACT	POI CONTACT	VICTIM CONTACT	WITNESS CONTACT
		d (SE) (95% CI)	d (SE) (95% CI)	d (SE) (95% CI)	d (SE) (95% CI)
Any emotional/Behavioural Problem		n.s	0.61 (0.18)*** (0.27 – 0.95)	n.s	n.s
	Anxious and fearful behaviour	-0.25 (0.13)* (-0.51 – 0.01)	n.s	n.s	n.s
	Aggressive behaviour	n.s	0.52 (0.19)** (0.14 – 0.90)	n.s	n.s
	Hyperactivity and inattention	n.s	0.46 (0.19) (0.09 – 0.83)**	n.s	n.s
Developmental Risk Profiles	Pervasive risk	n.s	1.30 (0.24)*** (0.83 – 1.77)	n.s	0.41 (0.19)* (0.05 – 0.78)
	Misconduct risk	n.s	0.59 (0.19)*** (0.22 – 0.95)	n.s	-0.27 (0.16)* (-0.59 – 0.05)
	Mild generalized risk	n.s	n.s	n.s	n.s

Note: Comparisons derived from tests of interaction as outlined by Altman and Bland (2003). Significant differences (d) indicate greater or lower effect for boys compared to girls.
n.s = non-significant differences among gender; *p<.05; **p<.01; ***p<.001
POI = person-of-interest
Emotional/behavioural problems were indexed using the anxious and fearful behaviour, aggressive behaviour, and hyperactivity and inattention subdomains of the emotional maturity domain of the Australian Early Development Census

© 2021 Dean K et al. *JAMA Network Open.*
